# Interactions of adolescent social experiences and dopamine genes to predict physical intimate partner violence perpetration

**DOI:** 10.1371/journal.pone.0172840

**Published:** 2017-03-06

**Authors:** Laura M. Schwab-Reese, Edith A. Parker, Corinne Peek-Asa

**Affiliations:** 1 Department of Community & Behavioral Health, College of Public Health, University of Iowa, Iowa City, Iowa, United States of America; 2 Department of Occupational & Environmental, College of Public Health, University of Iowa, Iowa City, Iowa, United States of America; Xi'an Jiaotong University School of Medicine, CHINA

## Abstract

**Objectives:**

We examined the interactions between three dopamine gene alleles (DAT1, DRD2, DRD4) previously associated with violent behavior and two components of the adolescent environment (exposure to violence, school social environment) to predict adulthood physical intimate partner violence (IPV) perpetration among white men and women.

**Methods:**

We used data from Wave IV of the National Longitudinal Study of Adolescent to Adult Health, a cohort study following individuals from adolescence to adulthood. Based on the prior literature, we categorized participants as at risk for each of the three dopamine genes using this coding scheme: two 10-R alleles for DAT1; at least one A-1 allele for DRD2; at least one 7-R or 8-R allele for DRD4. Adolescent exposure to violence and school social environment was measured in 1994 and 1995 when participants were in high school or middle school. Intimate partner violence perpetration was measured in 2008 when participants were 24 to 32 years old. We used simple and multivariable logistic regression models, including interactions of genes and the adolescent environments for the analysis.

**Results:**

Presence of risk alleles was not independently associated with IPV perpetration but increasing exposure to violence and disconnection from the school social environment was associated with physical IPV perpetration. The effects of these adolescent experiences on physical IPV perpetration varied by dopamine risk allele status. Among individuals with non-risk dopamine alleles, increased exposure to violence during adolescence and perception of disconnection from the school environment were significantly associated with increased odds of physical IPV perpetration, but individuals with high risk alleles, overall, did not experience the same increase.

**Conclusion:**

Our results suggested the effects of adolescent environment on adulthood physical IPV perpetration varied by genetic factors. This analysis did not find a direct link between risk alleles and violence, but contributes to growing research indicating that if genetic factors contribute to perpetration, this relationship is likely complicated and the result of interactions with other factors.

## Introduction

Intimate partner violence (IPV), defined as psychological, physical, or sexual abuse within the context of a current or former romantic relationship, is a substantial threat to health and well-being. Approximately one-in-three women and one-in-four men in the United States report experiencing physical or sexual IPV.[1] Much of the IPV literature focuses on victimization and the limited research on perpetrators, especially studies using longitudinal designs, has hampered efforts to develop and implement effective interventions for IPV perpetration.[2–4]

Literature suggests the etiology of IPV perpetration is multifactorial.[3] Static antecedents, stable characteristics that a relatively resistant to modification, are frequently studied as contributors to IPV perpetration. A recent systematic review found that demographic characteristics, including age, socioeconomic status, race/ethnicity, and marital status, and other static antecedents, including mental health/illness and personality, are significant predictors of IPV perpetration.[4] Distal antecedents, characteristics that are temporally removed from the time of perpetration but may indirectly contribute to behavior, have also been evaluated as potential contributors to IPV perpetration. Exposure to violence in the family of origin has been studied extensively and has consistently been shown to be associated with increased risk for adulthood IPV perpetration.[4, 5] Risky adolescent behaviors, including substance use and engaging with deviant peers, has also been associated with adulthood IPV perpetration.[5] Proximal antecedents, events or situations near the time of perpetration, may also contribute to IPV perpetration. Community factors, such as collective efficacy or social control, interpersonal factors, such as relationship discord or deviant peers, and individual factors, such as substance abuse, may also directly contribute to perpetration.[3, 4]

Multiple etiological frameworks have been developed to explain why IPV perpetration occurs.[3, 6] However, the contributions of genetic factors have generally not been considered in these frameworks, despite research supporting genetic contributions to other forms of aggression.[7, 8] To our knowledge, three studies have examined genetic contributions to IPV perpetration,[9–11] including only one that examined specific genes.[11] In that study, some variants of the Monoamine Oxidase A gene and the serotonin transporter gene were associated with increased odds of more frequent perpetration of IPV.[11] In light of the significant contributions of gene by environment interactions to other forms of aggression perpetration, the dearth of research on these interactions and IPV perpetration may be a significant limitation to knowledge of the etiology of IPV. To address this gap in the literature, the purpose of this analysis was to conduct a gene by environment analysis of three dopamine genes and two components of the social environment during adolescence to predict physical IPV perpetration among adults. Although genetic contributions are, at this point not modifiable, understanding the interaction between genetic factors and distal antecedents to IPV perpetration may begin to untangle the complex relationships between biological processes and environmental experiences. This type of knowledge may inform the development of interventions focused on altering adolescent social environments.

### Conceptual framework

This analysis is guided by the Catalyst Model of Aggression, which theorizes individuals develop a behavioral predisposition to perpetrate violence as a result of interactions between their early life experiences and genes.[12] As a result of this behavioral predisposition, individuals engage in a range of typical behavioral responses when they encounter stressful situations.[12] For this analysis, we focus on factors that contribute to the behavioral predisposition, specifically genes and adolescent experiences. Based on a comprehensive literature review, we focus this analysis an exploration of three dopaminergic genes and their interactions with exposure to violence and school social environment.

### Genetic polymorphisms

Dopamine is a neurotransmitter with significant influence on motivation and learning.[13] Individual processing of dopamine is influenced by dopaminergic polymorphisms,[14] defined as variances in a gene or DNA sequence that occur relatively frequently in the general population.[15] These polymorphisms vary from a change in one specific nucleotide base pair (single nucleotide polymorphisms) to a pattern of repeating base pairs (variable number tandem repeats).[15]

The dopamine transport gene (DAT1) is a variable number tandem repeat gene.[16] There are many different alleles, defined as the various forms of the gene, for the DAT1 gene.[16] The 10-repeat allele is associated with an efficient re-uptake process, which reduces the amount of available dopamine in the synapse.[14, 17] Research on the association between DAT1 polymorphisms and violence perpetration suggests the 10-repeat allele is associated with violent delinquency in adolescent men [13] and increased sensitivity to the environment when homozygous (two 10-repeat alleles).[18]

Two dopamine receptor genes (DRD2, DRD4) have been evaluated as potential contributors to violence.[19] DRD2 contains a single nucleotide polymorphism [20] and encodes the D2 subtype of dopamine receptors.[20] The A-1 allele of the DRD2 gene is associated fewer brain D2 dopamine receptors, which results in reduced receptor availability.[21] Carriers of at least one A1-allele may have a heightened response to stressful situations [18] which may contribute to the increased risk of violence perpetration.[13, 19] DRD4 contains a variable number of tandem repeats [22] and influences D4 dopamine receptors.[19] Carrying at least one 7-repeat allele is associated with diminished response to dopamine,[23] a range of aggressive behaviors,[19] and a differential response to the environment.[18]

### Early exposure to violence

Although much of the literature on exposure to violence during youth focuses on child maltreatment and parental IPV, there is some evidence to suggest exposure to violence in the community may also contribute to risk for IPV perpetration.[4, 5, 24, 25] Men who report exposure to violence in their neighborhood are more likely to perpetrate IPV compared with men who report no exposure to violence in their neighborhood and women who report exposure to neighborhood violence are more likely to be victims than their non-exposed peers.[24, 25] Additionally, violence within the adolescent social network is a significant predictor of later IPV perpetration.[24, 25] Young men from adolescent social networks with a high proportion of members who engaged in violent fights had a much higher probability of perpetrating IPV compared to men who had less violent social networks.[26] Additional research suggests that youth exposed to higher level of delinquency in their social networks are more likely to perpetrate IPV as adults.[27] In contrast to these findings, some research suggests that only physical childhood abuse and witnessing parental violence, not exposure to neighborhood violence, are linked to adulthood IPV perpetration.[28]

### School social environment

There has been limited investigation of the school social environment as a risk factor for adulthood IPV perpetration. However, improvements to the school social environment have reduced violence on campus [29] and overall violence outside of school.[30] Students exposed to unsupportive school social environments, an overall measure of the quality of interactions between students, their peers, and adults at school, are more likely to be victims and perpetrators of a range of violent behaviors compared to students at schools with more positive social environments.[31, 32] School social environment may also have indirect effects on violence in early adulthood as it may serve as a buffer between exposure to violence and violence perpetration during early adulthood.[33] These findings are important to IPV prevention efforts because adolescent violence perpetration is associated with an increased risk for IPV perpetration during adulthood.[34] If school social environment is significantly associated with adulthood IPV perpetration, interventions to help students feel safe and connected to social networks at school may long-term impacts on IPV perpetration.

### Study objective

Given the limited literature on the relationship of specific genetic polymorphisms to IPV perpetration, and the inconsistent or unknown relationships between adolescent experiences and IPV perpetration, the purpose of this analysis is to examine the role of dopamine genes, in combination with adolescent environment, in adulthood IPV perpetration. Using a nationally-representative prospective cohort study, we examine the probability of perpetrating IPV during adulthood based on three dopamine genes and their interactions with exposure to community violence and school social environment.

## Methods

### Study population

Participants for this analysis were enrolled in The National Longitudinal Study of Adolescent To Adult Health (Add Health). Within the initial sample, a subset of 20,745 adolescents in grades seven through twelve was purposefully sampled to be representative of the US population and participated in a prospective cohort study that surveyed participants in 1994–1995, 1996, 2001–2002, and 2008.[35] Systematic sampling methods and stratification were used to ensure the selected schools were representative of the overall US school.[35] Of the 20,745 participants enrolled in Wave I, a total of 15,701 participants (attrition rate: 24.3%) completed data collection during Wave IV, when participants were ages 24 to 32 years.

To be eligible for this analysis, participants must have completed Wave IV data collection, been in a relationship during the past twelve months, answered questions on the exposure, interaction, and outcome variables, and completed genotyping. Participants who were not in a relationship during the past twelve months were excluded because they reported on relationship experiences that occurred more than twelve months prior to the interview and research on survey methodologies has found that longer reporting periods are associated with underreporting and inaccuracy.[36] Approximately 3,320 (21.1%) participants were excluded because they did not have a romantic partner in the prior twelve months. Approximately 600 (3%) additional participants were excluded due to missing data for physical IPV perpetration, exposure to violence, or school social environment. As directed by the Add Health research team, participants who were excluded from the analysis were not removed from the dataset. Weights for excluded participants were set to 0.000001, which allows for the correct weighting process but does not include these participants in the estimates produced by the model.

This analysis was reviewed by The University of Iowa Institutional Review Board and deemed “not human subjects research” and was not subject to further review.

### Measures

#### Outcome

Physical intimate partner violence (IPV) perpetration was measured during Wave IV data collection (2008–2009).[35] Participants were asked to report, “How often (have/did) you (slapped/slap), hit, or (kicked/kick) [partner’s initials]?”.[35] Responses were dichotomized and participants were coded as perpetrators if they reported slapping, hitting, or kicking their partner at least once in the prior year.

#### Dopamine genes

The Add Health Study contained genetic data for all participants who consented to the biological specimen portion of the study. Additional information on the protocol, equipment, genotyping, and data cleaning for each gene was available elsewhere.[37] For this analysis, each dopaminergic polymorphism was categorized as associated with increased risk for violence (“risk allele”) or not associated with increased risk based on prior literature of the functional differences in alleles and associations with violence [13, 18, 19]. We categorized participants as at risk for each of the three dopamine genes using this coding scheme: two 10-R alleles for DAT1; at least one A-1 allele for DRD2; at least one 7-R or 8-R allele for DRD4. Although alternate methods of categorization of the genes were available, we chose to a priori categorization of the alleles due to the strong evidence of the associations of these risk categorizations with other forms of violence.

#### Interacting variables

Two types of adolescent experiences were included as potential modifiers of the relationships between dopaminergic polymorphisms and physical IPV perpetration: exposure to violence and school social environment.

Adolescent exposure to violence was measured during Wave I data collection in 1994–1995.[35] Participants reported how frequently they were exposed to the several different types of violence (i.e., threatened with knife or gun, shot, stabbed, beaten up, saw someone shot/stabbed, physical fight) in the twelve months prior to data collection [35]. Response options were “Never,” “Once,” and “More than once.”.[35] In our literature review, we did not find guidance on the treatment of these questions so we modeled the scores in multiple ways and selected the categorization based on the Akaike information criterion (AIC) value.[38] For the final categorization of the scale, we summed the number of types of violence experienced in the year prior to Wave I data collection when participants were in middle or high school.

During Wave I, participants answered a series of five questions on their perceptions of the school social environment.[35, 39] Responses to these questions were reported on a five-point, Likert-scale ranging from “strongly disagree” to “strongly agree”.[35] In this analysis, school social environment was the average score across the five questions. Scores ranged from 1 (“Strongly Agree”) to 5 (“Strongly Disagree”), where higher scores indicated a more negative perception of the school social environment.[39] This scale had good internal consistency (α = 0.79) in the Add Health sample.[39]

### Statistical analysis

We weighted and clustered the analysis by school and primary sampling unit to account for the complex survey design of the Add Health study. As a nationally-representative weighted analysis, the demographic characteristics of the sample were weighted to be similar to the demographic characteristics of population of adolescents in the United States during the mid-1990s. The distribution of polymorphisms and risk for physical IPV perpetration differed by race so we stratified by race to avoid bias associated with race-specific patterns of polymorphisms. After stratifying by race, the number of racial minority participants in each cell was too small to make meaningful interpretations. We also examined the potential differing relationships between dopamine genes and environmental exposure by gender and found no significant differences by gender.

We used bivariate analysis to estimate the distribution of exposure to violence, perceptions of school social environment, and each of the dopaminergic polymorphisms by gender and race. We conducted weighted, clustered simple logistic regression to determine if the dopaminergic polymorphisms, adolescent exposure to violence, or school social environment were associated with physical IPV perpetration. We also conducted weighted, clustered logistic regression including the main effect of dopaminergic polymorphisms and adolescent exposures, and the interaction effect of the two factors were used to determine if adolescent experiences moderated the relationship between dopamine polymorphisms and physical IPV perpetration. We presented the results of the moderation analyses as the odds ratio comparing each category (i.e., risk gene and high environmental exposure; risk gene and low environmental exposure; and non-risk gene and high environmental exposure) with a common reference group (i.e., non-risk gene and low environmental exposure) and also compared high environmental exposure to low environmental exposure within each strata of genes. To allow for visual comparison of these effects, we graphed the predicted probability for each of the combinations of risk.

## Results

### Adolescent exposures, polymorphisms, and perpetration of physical IPV

On average, participants were exposed to less than one type of violence (M = 0.5; 95% CI:0.4–0.5) during the twelve months prior to Wave I data collection (Table 1). Approximately 65% (95%CI: 63.0–66.9) experienced no types of violence, 20% (95%CI:19.1–21.8) experienced one form of violence, and 15% (95%CI:12.2–16.9) experience multiple forms of violence. For each additional type of violence exposure during adolescence, the odds of perpetrating physical IPV during adulthood increased by 41% (cOR:1.41, 95%CI:1.29–1.56).

**Table 1:** Estimated percent and 95% confidence intervals of adolescent exposure to violence, perceived school social environment, dopamine genes, and associations with physical IPV perpetration.

	Mean (95% CI) ^a^	Association with IPV^b^
Exposed to Violence^c^	0.5 (0.4–0.5)	1.41 (1.29–1.56)
School Social Environment^d^	2.3 (2.2–2.4)	1.25 (1.08–1.46)
	Estimated Proportion (95%CI) ^a^	Association with IPV^b^
**DAT1**		
2 risk alleles	55.7 (53.9–57.5)	0.95 (0.75–1.21)
0 or 1 risk alleles	44.3 (42.5–46.1)	Ref
**DRD2**		
1 or 2 risk alleles	38.1 (36.7–39.5)	1.05 (0.81–1.38)
0 risk alleles	61.9 (60.5–63.3)	Ref
**DRD4**		
1 or 2 risk alleles	37.0 (35.8–38.3)	1.05 (0.80–1.38)
0 risk alleles	63.0 (61.7–64.2)	Ref

^a^Estimated column percent and 95%CI

^b^Crude odds ratio and 95%CI predicting physical IPV perpetration

^c^Total number of types of violence exposures ranging from 0 to 6

^d^Participants’ mean score on a scale ranging from 1 to 5, where higher numbers indicate a more negative perception.

On a scale where “1” indicated a strongly connected school environment, “3” indicated a neutral option about the school environment, and “5” indicated a strongly disconnected school environment, the average score among participants was a 2.3 (95%CI:2.2–2.4) (Table 1). The median score was 2.1 (95%CI:2.0–2.1), the 25^th^ percentile was 1.8 (95%CI:1.7–1.9), and the 75^th^ percentile was 2.8 (95%CI:2.7–2.9). An increasingly disconnected perception of the school environment (referent group = one-point smaller mean score) was significantly associated with increased odds of physical IPV perpetration during adulthood (cOR:1.25; 95%CI:1.08–1.46).

More than half of participants (55.7%; 95%CI:53.9–57.5) were categorized as having homogeneous DAT1 risk alleles (Table 1). Approximately a third of participants had at least one DRD2 risk allele (38.1%; 95%CI:36.7–39.5) or at least one DRD4 risk allele (37.0%; 95%CI:35.8–38.3). None of the risk alleles were associated with significantly increased odds of physical IPV perpetration during adulthood.

### Interaction of polymorphisms and exposure to violence

**Fig 1:**
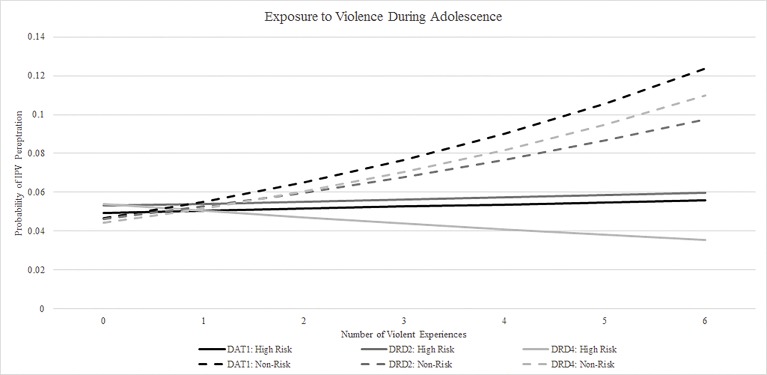
Predicted probability of perpetrating physical IPV, by polymorphism and adolescent exposure to violence.

Overall, higher exposure to violence during adolescence (referent group = exposed to one less type of violence) was associated with significantly increased odds of physical IPV perpetration among participants with no DAT1 or DRD4 risk alleles (Table 2). The relationship was similar, although not statistically significant, among individuals with no DRD2 risk alleles. In contrast, higher exposure to violence during adolescence was not associated with increased odds of physical IPV perpetration among individuals with high risk alleles. DAT1, DRD2, and DRD4 risk alleles were not significantly associated with physical IPV perpetration in the absence of exposure to violence. Graphical representation of the probability of perpetration visually clarified these complex relationships (Fig 1). Among those with non-risk alleles, the probability of perpetration tended to increase as the number of violence experiences increased. In contrast, the probability of perpetration stayed relatively flat among those with risk alleles as exposure to violence increased, which was supported by the lack of statistically significant differences in the odds of perpetrating physical IPV among individuals with risk alleles and high exposure to violence compared with individuals with risk alleles and low exposure to violence (Table 2).

**Table 2:** Associations between perpetrating physical intimate partner violence and the interaction of dopaminergic polymorphism and exposure to violence.

	High Exposure	Low Exposure	OR (95%CI) for exposure to violence within strata of gene^c^
	OR (95%CI)^a^	OR (95%CI)^b^	
**Exposure to Violence**			
**DAT1**			
2 risk alleles	1.22 (0.52–2.85)	1.07 (0.82–1.39)	1.14 (0.47–2.76)
0 or 1 risk alleles	**2.89 (1.04–8.04)**	Ref	
**DRD2**			
1 or 2 risk alleles	1.31 (0.49–3.48)	1.15 (0.86–1.54)	1.13 (0.41–3.18)
0 risk alleles	2.22 (0.94–5.29)	Ref	
**DRD4**			
1 or 2 risk alleles	0.79 (0.28–2.26)	1.23 (0.90–1.68)	0.64 (0.21–1.98)
0 risk alleles	**2.66 (1.10–6.40)**	Ref	
**School Environment**			
**DAT1**			
2 risk alleles	**2.44 (1.33–4.46)**	0.88 (0.54–1.45)	**2.76 (1.30–5.88)**
0 or 1 risk alleles	2.14 (0.78–5.84)	Ref	
**DRD2**			
1 or 2 risk alleles	1.85 (0.83–4.11)	**1.68 (1.04–2.70)**	1.10 (0.37–3.24)
0 risk alleles	**4.04 (1.86–8.79)**	Ref	
**DRD4**			
1 or 2 risk alleles	**2.02 (1.01–4.01)**	1.50 0.91–2.45)	1.35 (0.54–3.33)
0 risk alleles	**3.42 (1.55–7.56)**	Ref	

^a^Defined as highest level of possible exposure (i.e., exposed to six types of violence or very low school connectedness)

^b^Defined as lowest level of possible exposure (i.e., no exposure to violence or very high school connectedness)

^c^Comparion of high exposure to low exposure among individuals with same level of risk alleles.

### Interaction of polymorphisms and school social environment

**Fig 2:**
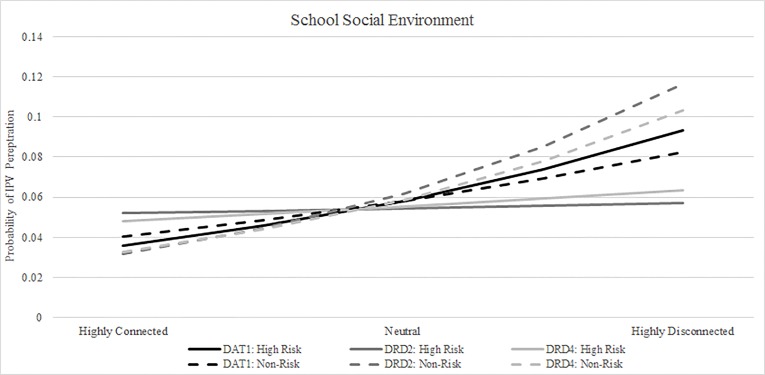
Probability of perpetrating physical IPV, by polymorphism and perception of school social environment.

In general, individuals with very low perceived school connectedness tended to have increased odds of perpetration physical IPV compared to individuals with very high school connectedness across levels of genetic risk (Table 2). However, among individuals with risk alleles for DRD2 or DRD4, decreasing school connectedness was not significantly associated with physical IPV perpetration (Table 2: OR Within Strata). Based on graphical representation of the probability of perpetration (Fig 2), individuals with high risk DRD2 or DRD4 risk alleles appear to have a reduced response to school social environment. Individuals with DRD2 or DRD4 risk alleles and low school connectedness did not have significantly increased odds of physical IPV perpetration compared with individuals with DRD2 or DRD4 risk alleles and high school connectedness (Table 2).

The relationship between DAT1 and school environment did not follow the same pattern as DRD2 and DRD4 (Table 2). In comparing individuals with non-risk alleles, individuals with low perceived school connectedness did not have significantly increased odds of physical IPV perpetration, compared with individuals with high perceived school connectedness. In contrast, among individuals with DAT1 risk alleles, low perceived school connectedness was significantly associated with increased odds of physical IPV perpetration, compared with individuals with high perceived school connectedness. In general, probability of perpetration among individuals with DAT1 risk alleles matched the probability of perpetration among individuals with DAT1 non-risk alleles to a greater extent than among analyses of DRD2 or DRD4 risk alleles (Fig 2).

## Discussion

The purpose of this analysis was to examine the relationship of dopamine genes and physical IPV perpetration, considering interactions with exposure to violence and perceived school environment. The results of these analyses suggested the etiology of physical IPV perpetration was complex and that, in some circumstances, dopamine polymorphisms were associated with differing probability of physical IPV perpetration. However, DAT1, DRD2, and DRD4 polymorphisms were not directly associated with odds of physical IPV perpetration.

In the first study of genes and IPV perpetration, a composite risk score composed of polymorphisms of the Monoamine Oxidase A gene and the serotonin transporter gene was associated with increased odds of more frequent perpetration of IPV.[11] In contrast to that study, our results suggested these dopamine polymorphisms did not directly contribute to the odds of perpetrating physical IPV during adulthood, but rather some polymorphisms interacted with environmental experiences (i.e., the effect of the polymorphism was different across different experiences) to contribute to physical IPV perpetration. This finding was consistent with prior findings on the potential contributions of these genes to overall aggression, which suggested that an increasing number of risk alleles had differential effects on violence perpetration dependent upon exposure to adverse environmental experiences [7]. However, previous findings on dopamine genes and other forms of violence have suggested individuals with high risk dopamine alleles tend to have an increased aggressive response to adverse environmental exposure.[7, 8] In contrast, our study found, in general, no significant increase in physical IPV perpetration among individuals with risk alleles and exposure to violence during adolescence or low perceived connectedness at school. It is unclear why these relationships differ and additional research is necessary to duplicate these findings among other samples. If replicated, these divergent findings may suggest the etiology of IPV perpetration differs from other forms of aggression or that dopamine processing plays a different role in IPV compared with other forms of violence and aggression.

As with all studies of the contribution of genetics to complex outcomes, it is important to consider the limitations of this study. First, this study uses self-reported physical IPV perpetration, which may not be the most reliable measure.[40] Second, this study is limited to a self-identified white sample because small cell sizes prevented meaningful investigation of these relationships among other groups. In addition, this type of gene by environment analysis is correlational, based on probabilistic methods, and does not imply a cause-and-effect relationship. Although these issues with gene by environment analyses are not necessarily limitations to the research when correctly interpreted within this framework, the interpretation should not be deterministic.[11] Additionally, researchers and practitioners must be mindful of the ethical implications, including the possibility of stigma associated with incorrect interpretation of the results. Finally, this analysis is not able to account for epigenetic differences, which play an important role in phenotypic plasticity resulting from the interaction between the genome and the environment.

Despite these limitations, our findings, combined with the results of current and future studies of the genetic contributions to IPV, may have important implications for future research and practice. Gene-environment studies may contribute to a more comprehensive understanding of IPV perpetration and may further clarify the role of neurotransmitters. Ultimately, it may be possible to develop pharmacological or behavioral intervention strategies based upon constellations of genetic and environmental risk factors. However, additional research is necessary to further clarify these relationships before this line of research will have practical applications to intervention development and implementation.
